# The Anxiolytic Effect of Midazolam in Third Molar Extraction: A Systematic Review

**DOI:** 10.1371/journal.pone.0121410

**Published:** 2015-04-07

**Authors:** Qi Chen, Lufei Wang, Lina Ge, Yuan Gao, Hang Wang

**Affiliations:** State Key Laboratory of Oral Diseases, Department of Oral and Maxillofacial Surgery, West China Stomatology Hospital, Sichuan University, Chengdu, Sichuan, China; Central South University, CHINA

## Abstract

**Purpose:**

To assess the efficacy of midazolam for anxiety control in third molar extraction surgery.

**Methods:**

Electronic retrievals were conducted in Medline (via PubMed, 1950-2013.12), the Cochrane Central Register of Controlled Trials (CENTRAL) (The Cochrane Library 2013, Issue 3), Embase (via OVID 1974-2013.12), and the System for Information on Grey Literature in Europe (SIGLE). The bibliographies of relevant clinical trials were also checked. Randomized controlled trials satisfying the inclusion criteria were evaluated, with data extraction done independently by two well-trained investigators. Disagreements were resolved by discussion or by consultation with a third member of the review team.

**Results:**

Ten studies were included, but meta-analysis could not be conducted because of the significant differences among articles. All but one article demonstrated that midazolam could relieve anxiety. One article demonstrated that propofol offered superior anxiolysis, with more rapid recovery than with midazolam. Compared with lorazepam and diazepam, midazolam did not distinctly dominate in its sedative effect, but was safer. Two articles used midazolam in multidrug intravenous sedation and proved it to be more effective than midazolam alone.

**Conclusion:**

It was found, by comparison and analysis, that midazolam might be effective for use for anxiety control during third molar extraction and can be safely administered by a dedicated staff member. It can also be used with other drugs to obtain better sedative effects, but the patient’s respiratory function must be monitored closely, because multidrug sedation is also more risky.

## Introduction

Dentistry and anxiety have always been inextricably linked, particularly with regard to third molar extractions, which provide a relatively intense surgical stimulus. Anxiety toward dental procedures varies from a suppressed fear of pain to a phobia. Patients may also have symptoms such as sweating, tremors, arrhythmias, and vasovagal reactions, which may make treatment difficult or even impossible. Thus, sedative measures have attracted serious attention among dental practitioners and researchers. “Minimal sedation” or “anxiolysis” is a drug-induced condition in which patients respond normally to verbal commands, while the cardiovascular systems and spontaneous breathing are unaffected. Cognitive function and coordination may be impaired [1].

Midazolam, as a benzodiazepine, has both anxiolytic and amnesic properties. It has become increasingly popular as an anesthetic modality for managing the apprehensive dental patient [2–4]. In addition to midazolam, other benzodiazepines, opioids, and barbiturates are among the most common drug classes included in intravenous sedation regimens [5]. Pharmacologically, these drugs depress specific areas in the central nervous system that control pain and anxiety.

Though some patients require sedation to make tooth extraction more tolerable, the precise effects of midazolam remain unclear, due to incremental increases in anxiety. Therefore, we reviewed the selected articles to determine the efficacy and safety of midazolam used in anxious patients during third molar extraction and compared them with other regimens of sedative drugs to determine the incidence of common adverse drug reactions.

## MATERIALS AND METHODS

### Inclusion and exclusion criteria

This study was approved by the Ethics Committee of the Sichuan University, Chengdu, China. Two reviewers independently assessed the title and abstract of each retrieved citation for eligibility according to the following inclusion criteria: (1) Studies had to include dentally anxious outpatients who had undergone third molar extraction, regardless of gender or race; (2) published studies, including grey literature, had to be randomized, double-blind, and refer to midazolam’s effect on dental anxiety compared with that of placebo or other anti-anxiety agents; (3) and studies had to have outcome indices, including the patients’ anxiety levels. Studies were excluded if they met the following criteria: (1) Descriptive research such as reviews, case reports, and clinical observations were excluded, as were basic experiments; and (2) studies including patients younger than 18 years old were excluded. For all potentially eligible citations, we retrieved the full-text article, which was assessed independently by two reviewers. Disagreements were resolved by discussion or by consultation with a third member of the review team.

### Identification of studies

Two reviewers independently searched for all publications using electronic databases, including the Cochrane Central Register of Controlled Trials (CENTRAL) (The Cochrane Library 2012, Issue 3), Medline (via PubMed 1950–2012.12), Embase (via OVID 1974–2012.12), and SIGLE (System for Information on Grey Literature in Europe). The following search terms were used to search Medline and CENTRAL: “midazolam”, “tooth extraction”, “tooth extractions”, “anxiety”, “third molar”, “third molars”,”wisdom tooth”, and “wisdom teeth”. These terms were modified to search Embase and other databases as required. The search strategy was combined with the highly sensitive strategy developed by the Cochrane Collaboration for identifying RCTs in Medline: sensitivity maximizing. We also searched the reference lists of the identified studies.

### Data extraction and bias assessment

We produced a form for data extraction including population, study design, treatment, and efficacy assessment for each study (Table 1). Qualities of the included studies were assessed by a customized form for assessing risk of bias (Table 2), according to Cochrane Handbook 5.0. Any disagreement was resolved by consensus among all authors.

**Table 1:** Characteristics of included studies.

	Participants	Methods	Interventions	Efficacy assessment
Eberhart	T:50/50	Double-blinded	T:0.05mg/kg midazolam	·Erlanger anxiety and tension scale
(2000)	C:50	RCT	1.5g/kg clonidine ivgtt	(1) Pre-treat: 34 vs 34 vs 33
[7]			C:none	Post-surg: 29 vs 29 vs 29
			·Follow-up time: one day	(2) P >0.50
				·postoperative effect
Leitch	T:55	Partially-blinded	Midazolam: Operator-controll	·VAS(0-100mm) Reduction
(2004)	C:55	RCT	ed anti-anxiety	(1) Propofol 21(SD 21) mm
[8]	Average age:		Propofol: Patient-maintained	Midazolam 11(SD 18) mm
	28± 6.5 y		anti-anxiety	(2) P = 0.01
				·DSST,time,satisfactory
Bell	T: 20/20/20	RCT	Titrated increments of 1 or 2	·Modified corah anxiety scale(1–5)
(2000)	C: 20		mg/min until anti-anxiety	(1) Sedated group -2.42(SD 3.5) vs
[9]			·Follow-up time: 2 weeks	control group 1.00(SD2.4)
			later	(2) P< 0.05
				·memory, HR, BP
Dionne	T: 199/194/	Double-blinded	T: midazolam/m+m/fentanyl	·Median cognitive anxiety(0–42)
(2001)	185/202	RCT	+m/m+f+methohexital	(1) P< 0.05
[10]	C: 205		C: saline	·pain, memory, alertness, movement
			·Follow-up time:24h after	verbalization, side-effect
			surgery	
Van der	T: 20	Double-blinded	Lorazepam(0.05mg/kg,M 3mg),	·Anxiety(0–3)
Bijl	C: 20/20	RCT	Diazepam(0.25mg/kg, M 20mg)	(1) P = 0.02
(1991)			midazolam(0.1mg/kg, M 8mg)	·Side-effect, pegboard test, amnesia
[11]			·Follow-up time: 2 hours later	
Van der	T: 25	Double-blinded	T:0. 1mg/kg midazolam	·Anxiety(0–3)
Bijl	C: 25	RCT	C: 10ml saline	(1) P = 0.02
(1987)	Average age:		·Follow-up time: one week	·Side-effect, pegboard test, memory
[13]	T: 23.84±4.97		later	alertness
	C: 23.96±9.55			
Jeries	T: 20	Double-blinded	T: 7.5mg midazolam	·salivary cortisol measurements
(2005)	C: 18	RCT	C: placebo	(1) P = 0.01
[14]			·Follow-up time: one month	·HAD scale
			post-surgery	
Milgrom	T:40/38/41/38	Double-blinded	T:midazolam/m+m/fentanyl+	·Median cognitive anxiety(0–42)
(1994)	C:50	RCT	m/f+m+methohexital	• Acceptance, movement
[15]	Average age:		C:Saline	verbalization, discomfort
	25.7± 5.3 y		·Follow-up time:24h after	
			surgery	
Studer	T: 12	Double-blinded	T: 7.5mg midazolam	·VAS(0-100mm)
(2012)	C: 12	RCT	C: 150μg clonidine	At the end of the surgery
[16]	Average age:		·Follow-up time: one week	(1) Midazolam 20mm
	24.4y		later	Clonidine 15mm
				(2) P = 0.61
Pereira-	T:14	Double-blinded	T: 7.5mg midazolam	·salivary cortisol measurements
Santos	C:14	RCT	C: 50% N_2_O	Midazolam p< 0.05
(2013)				N_2_O p > 0.50
[17]				

VAS: visual analogue scale RCT: randomized controlled trial Ivgtt: intravenously guttae

DSST: Digit Symbol Substitution test T: treatment group C: control group

HR: heart rate BP: blood pressure

**Table 2:** Customized form for assessing risk of bias of included studies.

Study	Sequence generation	Allocation concealment	Blinding	Incomplete outcome data	Selective reporting	Other bias	Level
Eberhart	-	-	+	+	+	+	C
(2000)							
[7]							
Leitch	+	+	-	+	+	?	C
(2004)							
[8]							
Bell	+	?	+	+	+	?	B
(2000)							
[9]							
Dionne	+	+	+	?	+	+	B
(2001)							
[10]							
Van der	?	?	+	+	+	+	B
Bijl							
(1991)							
[11]							
Van der	-	-	+	+	+	?	C
Bijl							
(1987)							
[13]							
Jeries	+	+	+	+	+	?	B
(2005)							
[14]							
Milgrom	+	?	+	?	+	+	B
(1994)							
[15]							
Studer	+	+	+	+	+	+	A
(2012)							
[16]							
Pereira-Santos	+	?	?	+	+	+	B
(2013)							
[17]							

+ = Proper

- = Improper

? = Unclear

^A^: low risk of bias

^B^: middle risk of bias

^C^: high risk of bias

### Statistical analysis

The Effect Size (ES) is calculated as the ratio of the mean difference between treatment and placebo relative to the standard deviation (SD) of that difference:

ES = Mean of difference between treatment and placebo/SD of difference between treatment and placebo.

The ES is defined as small (0.2), medium (0.5), or large (0.8) [6] and is calculated with Systat, based on paired *t-*tests. Unlike *t-*test statistics, however, ES aims to estimate a population parameter, so it is not affected by sample size. The larger the ES, the more effective the treatment.

## RESULTS

### Literature search

**Fig 1:**
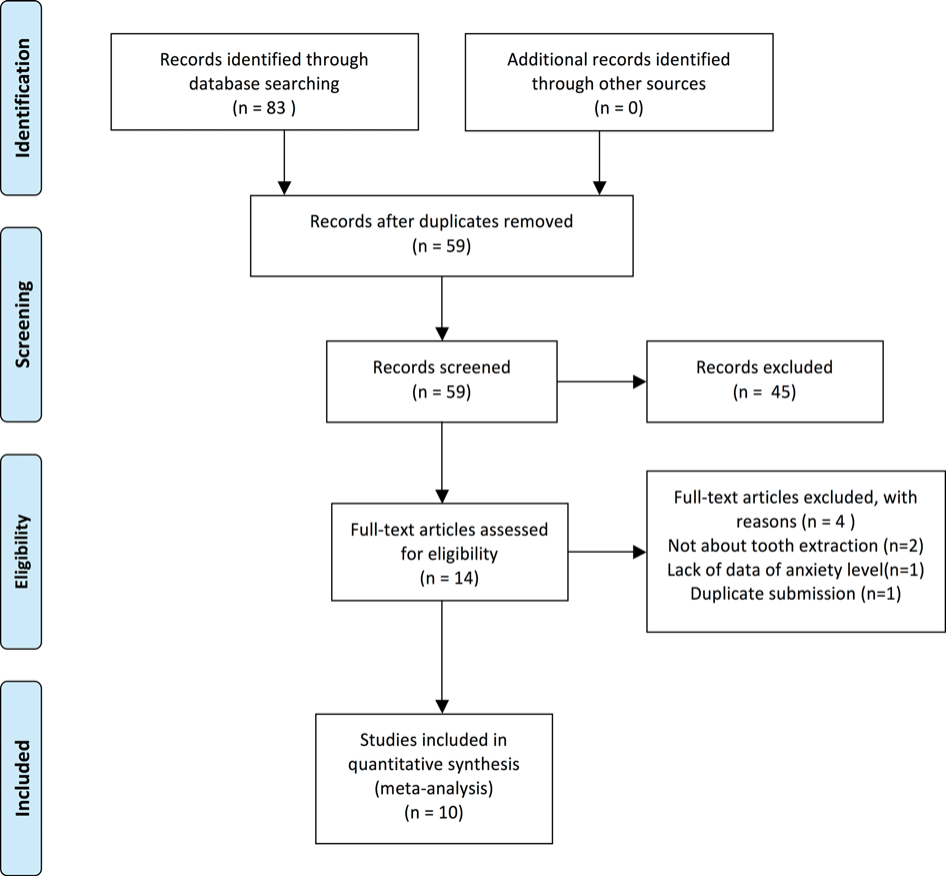
Flow Diagram for Study Search and Inclusion.

We identified 10 randomized clinical trials (RCTs) that form the basis of this review. Nine were double-blinded RCTs, and one was a partially blinded RCT. From 6 of these 10 articles, data comparing midazolam with placebo were retrieved. Information about midazolam compared with other sedative drugs (clonidine, propofol, lorazepam, and diazepam) could also be retrieved. These sedative drugs were given to patients intravenously or orally. To assess patients’ anxiety levels, investigators in these trials used different questionnaires, scales, and salivary cortisol measurements. A particularly important type of diversity existed in the comparisons being made by the primary studies, so meta-analysis was not possible (Fig. 1).

### Efficacy of midazolam compared with placebo in third molar extraction surgery

Except for one study, all others showed that midazolam had a statistically significant difference in anxiety relief when compared with placebo. After analysis, we found numerous shortcomings in research design in the only article in which contradictory results were reported (Table 1), and this study was rated ‘C’ (Table 2) on the customized form for assessing risk of bias and reporting quality of included studies. Sufficient statistics could be retrieved from three articles for ES calculation (Table 3), and all ES numbers exceeded 0.8, which further proved the effectiveness of the treatment. The incidence of adverse events when midazolam was used was no higher than when placebo was used. Therefore, for the ASA (American anesthesia association) 1 or 2 patients included in these studies, midazolam can be used as a safe and effective drug for anxiety control in third molar extraction surgery.

**Table 3:** Midazolam anti-anxiety compared with placebo

Study	Placebo	Midazolam	Sample size	ES
Dionne	Five minutes	Five mimutes	205 vs 199	3.87
(2001)[10]	14.13(SD 0.53)	12.23(SD 0.46)		
	Completion	Completion		
	12.60(SD 0.46)	10.38(SD 0.49)		
Bell	1.00(SD 2.4)	-2.42(SD 3.5)	20 vs 60	1.05
(2000)[9]				
Jeries	Intra-operatively	Intra-operatively	18 vs 20	5.79
(2005)	26.6(SD 2.0)	15.9(SD 1.7)		7.34
	Recovery room	Recovery room		
	29.5(SD 1.4)	18.0(SD 1.7)		

### Efficacy of midazolam in third molar extraction surgery compared with that of other drugs

In a single-blind controlled study [8] of patient-maintained anti-anxiety achieved with propofol compared with operator-controlled midazolam, 110 patients with third molar extraction were recruited for recordings. In the propofol group, the patients were allowed to titrate their sedation with propofol using TCI technology until they were ready to receive their local anaesthetic injection. In addition, this group was able to continue to titrate sedation throughout the surgery. The anxiety level was measured by VAS recordings taken after anti-anxiety and before surgery. The propofol patients showed a greater mean anxiety reduction of 21 mm (SD 21 mm), while midazolam patients had a mean reduction of 11 mm (SD 18 mm) (*p* = 0.010) [8]. Both techniques were well-tolerated and safe. Propofol sedation offered superior anxiolysis, quicker recovery, and less amnesia. In a randomized double-blind study with intravenous midazolam (0.1 mg/kg, maximum of 8 mg), diazepam (0.25 mg/kg, maximum of 20 mg), or lorazepam (0.05 mg/kg, maximum of 3 mg) for anti-anxiety, 60 patients of both genders, ranging in age from 17 to 32 years (median, 21 years), were included. Patients receiving lorazepam, diazepam, or midazolam showed significantly different improvements (*p* = 0.02) at 10 minutes post-administration, with greatest improvement in the diazepam group. There were no statistically significant differences among the three groups on arrival in the recovery room. Compared with the other two groups, patients in the diazepam group reported pain on injection [11].

### Midazolam used in multidrug intravenous anti-anxiety compared with midazolam only

Two double-blind controlled studies [10,15] examined 4 drug combinations (midazolam, midazolam-midazolam, fentanyl-midazolam, and fentanyl-midazolam-methohexital). One study [15] included 207 participants (57% women, mean age 25.7 ± 5.3 years), and another [10] included 985 participants (532 men, 453 women). Two additional articles [18,19], similar to the two included articles, were excluded, but their data were useful. All the articles suggested that the 4 drug combinations had increasingly greater anxiolytic effects, especially the fentanyl-midazolam and fentanyl-midazolam-methohexital groups, while the administration of additional midazolam during the procedure did not relieve anxiety more than the use of only midazolam before surgery. Transient respiratory depression occurred in patients in the two groups using fentanyl, but no other physiological changes were detected.

## DISCUSSION

Third molar extraction is a comparatively complicated procedure and commonly causes discomfort and anxiety for patients. Today, however, with the advances in dental research, people prefer, and have come to expect, more comfortable therapeutic procedures. Therefore, dental practitioners worked to find ways to relieve patient anxiety during surgery and identified the drug benzodiazepine, which has both anxiolytic and amnesic properties [12]. One of the benzodiazepines, midazolam, has a short half-life of 1.3–2.2 hours [20], and its metabolites have little pharmacological activity. It was first introduced and applied for anti-anxiety in dental clinical treatment in the 1980s [21]. Since then, midazolam has been widely used in third molar extraction surgery, and its anxiolytic effects have been studied. However, no systematic review of these studies has been conducted, and no conclusions have been drawn regarding the sedative effects of midazolam.

### The efficacy and safety of midazolam

Of the 10 articles included in our systematic review, only one concluded that midazolam was restricted to decreased anxiety during surgery [7]. This article lacked sequence generation and allocation concealment, which led us to believe that comparatively severe bias contributed to its results. Based on the results of the other 9 articles, our review provided evidence that midazolam is more likely to be efficacious in relieving anxiety during third molar extraction surgery. From these articles, we concluded that the route of medication was changing. Midazolam could be given through oral (0.25–0.5 mg/kg) or nasal (0.2 mg/kg) administration. Intramuscular injection was the fastest working method, but the disadvantages included pain at the injection site and the patient’s lack of ability to control the degree of anti-anxiety. Currently, bolus intravenous anti-anxiety (BIVS) and intravenous continuous infusion are primarily used. BIVS can be conducted by the anesthetist or the dentist, and the dose differs according to the patient’s physique. Compared with BIVS, intravenous continuous infusion produces better sedative and amnestic effects, but requires longer recovery time. The recommended speed of drug administration is 0.4 mg/kg/h. When we considered the safety of midazolam, we found that complications appeared to be transient, following the same time-course of therapeutic effects in general. Drowsiness, lack of coordination, disorientation, and decreased saturation of blood oxygen were the most common adverse events reported in the literature [10], leading us to recommend the continuous monitoring of oxygen concomitant with the intravenous application of midazolam; a continuous insufflation of oxygen should be included.

Analysis of data from these documents also showed that diazepam had the best sedative effect when compared with that of midazolam and lorazepam. However, diazepam often caused pain on injection and venous thrombosis; furthermore, it prolonged anti-anxiety because of its active metabolites with long plasma half-lives [13]. When we considered both efficacy and safety, we concluded that midazolam might be the best benzodiazepine to be used for anti-anxiety.

Further, the studies revealed that benzodiazepine, opioid, and ultra-short-acting barbiturate combinations had sedative effects superior to those obtained with midazolam alone but with increased risk, most notably associated with significant respiratory depression. To guarantee patient safety, we should continuously monitor the patient’s respiratory function, coupled with the practitioner’s experience and training, along with the equipment and drugs necessary to manage this complication. Now recent research verified that capnography [22] can provide noninvasive monitoring of ventilation and detect apnea during sedation, which should be spread to prevent serious complication during surgery.

### The amnesic effect of midazolam

Many previous investigations revealed the amnesic effects of midazolam. After review of our included literature, we found that although amnesia following the use of midazolam may be well documented by picture or object recall, incomplete amnesia was reported for operations. Thus, the effect of midazolam on the patient’s ability to forget pictures, objects, or a list of words cannot be directly related to clinical events [9]. It has also been shown that the plasma concentrations of midazolam determined the degree of amnesia [23].

### The method for measuring anxiety

The most important factor, which made meta-analyses impossible in our review, is that many anxiety scales were used. Data acquired from these scales could be either continuous variables or categorical variables, so it was difficult for us to merge them. In addition, some of the scales surveyed anxiety levels from the patients’ perspective, and some through the perspective of observers. Neither patients nor observers can accurately measure anxiety levels because of the influence of subjective factors. Two included studies [14,17] introduced a new technique whereby salivary cortisol was used to measure the level of anxiety. We know that free cortisol in plasma increases when people are anxious, and measurement of cortisol concentrations in saliva closely reflects the concentration of free cortisol in plasma [24,25]. Saliva collection may, therefore, be the preferred way to provide cortisol measurements. One study [18] showed that the use of both midazolam and nitrous oxide produced effective and safe sedation in anxious patients undergoing mandibular third molar extraction, as evaluated according to the VAS scale, but 7.5 mg of midazolam provided more effective sedation for reducing the salivary cortisol level, which may indicate that salivary cortisol levels can reveal anxiety levels with more sensitivity. We recommend that this method be widely adopted, to make the various anxiety measurements comparable.

### Limitations of our systematic review

Our review had shortcomings, since meta-analyses could not be performed because of the severe heterogeneity of the articles studied. We will continue to pay close attention to further research about the anxiolytic effect of midazolam and update our results. Finally, we call on more researchers to focus on unifying the methods of measuring anxiety and to search for more accurate and quantitative methods.

## CONCLUSIONS

The ideal sedative drug, which we believe to be suitable for use as a local anesthetic, should be reliable for anti-anxiety and have minimal effects on circulation, respiration, and recovery. The selected studies provided evidence that midazolam is efficacious for anxiety control. To ensure that the anxiolytic process is safe, we must evaluate the general condition of patients. Midazolam may come into wider use not only as an anxiolytic drug in third molar extraction surgery, monitoring of respiratory function using capnography may further increase safety during midazolam use as a sedative-hypnotic.

## Supporting Information

S1 PRISMA Checklist PRISMA 2009 checklist. (PDF)
